# Potential anti-inflammatory, antioxidant and antimicrobial activities of *Sambucus australis*

**DOI:** 10.1080/13880209.2017.1285324

**Published:** 2017-02-06

**Authors:** Jhéssica Benevides Bahiense, Franciane Martins Marques, Mariana Moreira Figueira, Thais Souza Vargas, Tamara P. Kondratyuk, Denise Coutinho Endringer, Rodrigo Scherer, Marcio Fronza

**Affiliations:** a Programa de Pós-Graduação em Ciências Farmacêuticas, Universidade Vila Velha, Vila Velha, Espirito Santo, Brazil;; b Department of Pharmaceutical Sciences, Daniel K. Inouye College of Pharmacy, University of Hawaii at Hilo, Hilo, HI, USA

**Keywords:** Natural products, phenolic compounds, nitric oxide, cytokines, nuclear factor-κB, macrophages

## Abstract

**Context:***Sambucus australis* Cham. & Schltdl. (Adoxaceae) is used in Brazilian folk medicine to treat inflammatory disorders.

**Objective:** To evaluate the *in vitro* anti-inflammatory, antioxidant and antimicrobial properties of *S. australis*.

**Materials and methods:** The anti-inﬂammatory activity of ethanol extracts of the leaf and bark of *S. australis* (1–100 μg/mL) were studied in lipopolysaccharide/interferon γ stimulated murine macrophages RAW 264.7 cells (24 h incubation) by investigating the release of nitric oxide (NO) and tumour necrosis factor-alpha (TNF-α) and in the TNF-α-induced nuclear factor kappa (NF-κB) assay. Minimum inhibitory concentration (MIC) was determined by the microdilution test (24 h incubation). Antioxidant activity was determined by 2,2-diphenyl-1-picrylhydrazyl (DPPH), ferric reducing antioxidant power (FRAP) and the NO scavenging assays. Chemical composition was assessed by LC-MS/MS.

**Results:** Antioxidant activities in the DPPH (IC_50_ 43.5 and 66.2 μg/mL), FRAP (IC_50_ 312.6 and 568.3 μg/mL) and NO radical scavenging assays (IC_50_ 285.0 and 972.6 μg/mL) were observed in the leaf and bark ethanol extracts, respectively. Solely the leaf extract showed significant inhibition of NO and TNF-α production in RAW264.7 cells at concentrations of 2 and 100 μg/mL, respectively, and suppression of TNF-α inhibition of NF-κB by 12.8 and 20.4% at concentrations of 50 and 100 μg/mL, respectively. The extract also exhibited antibacterial activity against *Salmonella typhimurium* (MIC 250 μg/mL) and *Klebsiella pneumoniae* (MIC 250 μg/mL). LC-MS/MS revealed the presence of chlorogenic acid and rutin as major compounds.

**Discussion and conclusion:** The results indicate that the ethanol leaf extract of *S. australis* exhibit prominent anti-inﬂammatory effects.

## Introduction

Inflammation is the immune system’s response to ward off injurious stimuli such as pathogens, damaged tissues or irritants. Both acute and chronic inflammation play essential roles in the restoration of homeostasis (Sacca et al. 1997; Serhan et al. 2007; Ricciotti & FitzGerald 2011). In inflammatory processes, activated macrophages secrete excessive nitric oxide (NO), prostaglandin E2 (PGE_2_) and pro-inflammatory cytokines, such as tumour necrosis factor-alpha (TNF-α) and interleukins (ILs). Pro-inflammatory mediators, particularly the inducible nitric oxide synthase (iNOS) for nitric oxide production, and cyclooxygenase (COX-2) for prostaglandin production, have demonstrated promotion/progression of various human inflammatory diseases (Zamora et al. 2000; Wright et al. 2010).

Nonsteroidal anti-inflammatory drugs (NSAIDs) are the most important group of available medicines used for the management of pain, and exhibit various anti-inﬂammatory, antipyretic and analgesic activities. However, the majority of NSAIDs also exhibit serious adverse effects, particularly gastrointestinal complications (Lichtenstein et al. 1995; Mckellar et al. 2007). Thus, the development of new drugs for the treatment of chronic inﬂammation and pain continues to be an issue of high interest, prompting a continuous search worldwide, for new compounds with anti-inflammatory properties and fewer side effects. Due to the properties claimed, and the needs of the pharmaceutical market, anti-inflammatory agents originating from natural products used in traditional medicine are a major focus of interest of the pharmaceutical industry (Akkol 2012; Russo et al. 2015).

*Sambucus australis* Cham. & Schltdl. (Adoxaceae), a shrub that is widespread in South America, is commonly known as *sabugueiro* in Brazil. Its aerial parts have been used in folk medicine to treat respiratory and inﬂammatory diseases, as a mild laxative, for diuretic and antipyretic purposes, in infusions and decoctions, and as a poultice (Brandão et al. 2006; Nunes et al. 2007; Nascimento et al. 2014). The main reported secondary metabolites for this species are flavonoids (kaempferol and quercetin), quercetin glycosides (rutin, hyperoside, isoquercetrin), triterpenes (ursolic acid), volatile oils, and phenolic acids (Lorenzi & Matos 1985; Scopel et al. 2010; Rao et al. 2011).

The biological activities of *S. australis* are practically unknown even through studies on other species of the *Sambucus* genus have been published. The potential anti-inﬂammatory activity of *Sambucus* genus was reported by Schwaiger et al. (2011), demonstrating that the leaf extract of *Sambucus ebulus* L. and its major isolated compound, ursolic acid, inhibited TNF-α production in human umbilical vein endothelial cells (HUVECs) and induced the expression of VCAM-1 and ICAM-1. Methanolic extract of *S. ebulus* was reported to exhibit antibacterial activity against methicillin resistant *S. aureus* (Salehzadeh et al. 2014) and displayed remarkable wound-healing activity (Süntar et al. 2010). Potential anti-inflammatory activities were also reported using an aqueous extract from the flower of *Sambucus nigra* L. (Harokopakis et al. 2006). The authors showed that the flower extract inhibits macrophage release of proinflammatory cytokines and suppresses the activation of neutrophils. These effects could be attributed to suppression of nuclear transcription factor κB activation and inhibition of phosphatidylinositol 3-kinase (Harokopakis et al. 2006).

Based on these promising biological activities described in other species of *Sambucus* genus, and the lack of biological studies with *S. australis* naturally found in Brazil, the purpose of this study was to evaluate the *in vitro* anti-inflammatory, antioxidant, and antibacterial activities of *S. australis.*

## Materials and methods

### Reagents

Interferon gamma (IFN-γ), lipopolysaccharide (LPS), penicillin, streptomycin, 2,2-diphenyl-1-picryl-hidrazyl (DPPH), butylated hydroxytoluene (BHT), triphenyl tetrazolium chloride (TTC), nitroprusside, TPTZ (2,4,6-tris(2-pyridyl)-s-triazine), chlorogenic acid, gallic acid, quercetin, rutin and Folin-Ciocalteau reagent were purchased from Sigma Chemical Co. (St. Louis, MO). Reporter lysis buffer and the luciferase assay system were purchased from Promega (Madison, WI). TNF-α ELISA kit was purchased from eBioscience (San Diego, CA). All other solvents and reagents were of analytical grade, and were purchased from Vetec and Dinamic (Rio de Janeiro, Brazil).

### Cell lines

Swiss 3T3 albino mouse fibroblasts (American Type Culture Collection – ATCC^®^ CCL-92^TM^), murine hepatoma (Hepa 1c1c7) cells (ATTC^®^ CRL-2026^TM^), mouse macrophages RAW 264.7 (ATCC^®^ TIB-71™) (Cell Line Service, Rio de Janeiro, Brazil), human embryonic kidney cells 293 (Panomic, Fremont, CA) were maintained in Dulbecco’s modified Eagle’s medium (DMEM) supplemented with 10% foetal bovine serum (FBS), 100 IU/mL penicillin and 100 μg/mL streptomycin, at 37 °C, in a humidified atmosphere containing 5% CO_2_ (all Sigma).

### Plant material

Leaves and bark of *Sambucus australis* were collected in Tucunduva city, South Brazil, in March 2014 (Latitude −27.634394 and Longitude −54.408549). The plant was taxonomically identified by botanist Ms. Solange Zanotti Schneider, and a voucher specimen was deposited in the herbarium of the University Vila Velha/UVV (UVVES-2397).

### Preparation of plant extracts

The air-dried, ground leaves and bark (100 g) were first defatted with hexane (1 L) and then exhaustively extracted with ethanol (1 L) using a Soxhlet apparatus. Subsequently, the solvent was removed under vacuum at 40 °C (Fisaton 801, São Paulo, Brazil) and the ethanol leaf and bark extract (12.7 and 3.5 g, respectively) were obtained. The extracts were stored at −20 °C until use.

### Determination of total phenolics

Total phenolic contents (TPC) in the extracts were estimated by the spectrophotometric Folin–Ciocalteu method, according to the Scherer and Godoy (2009) method. All analyses were performed in triplicate and the results are expressed as mean ± standard deviation. The TPC is expressed as milligrams of pyrogallol equivalents per gram of crude extract.

### Determination of tannin contents

Tannin content in the extract was estimated using the insoluble polyvinyl polypirrolidone (PVPP) method, as previously described (Singh et al. 2012). The plant extract solution was prepared at 1% in methanol, and then aliquots of 1 mL were mixed with 100 mg PVPP, vortexed, kept for 10 min at 4 °C, and centrifuged for 10 min at 800*g*. The non-tannin phenolic content was determined in the clear supernatant in a similar manner to the total phenolic content (Scherer & Godoy 2009). The tannin content was estimated as the difference between total phenolic and non-tannin phenolic content in the extract. All analyses were performed in triplicate and the results are expressed in milligram of pyrogallol equivalents per gram of crude extract.

### LC-ESI-MS/MS analyses

The identification of major chemical constituents in the ethanol leaf extract of *S. australis* was based on Zhu et al. (2012), with modifications. The chromatographic analyses were performed in a liquid chromatograph (Agilent 1200 series, Santa Clara, CA) coupled with triple quadrupole mass spectrometer detector (Applied Biosystems API 3200, Foster City, CA) with electrospray ionization (LC-ESI-MS/MS). The data were processed using the Analyst™ Software (version 5.0, Foster City, CA). All separations were performed on a Water Cortecs C18 column (Milford, MA), (150 mm ×4.6 mm, 2.7 μm) at 25 °C. The mobile phase consisted of an aqueous solution with formic acid (1% v/v) (A) and methanol with formic acid (1% v/v) (B) using a gradient elution at 0.6 mL/min of 10–50% B in 0–8 min, 50–70% B in 8–12 min, 70–10% B in 12–15 min, and conditioning time of 5 min. The samples were diluted with methanol at a concentration of 0.5 mg/mL. The compounds were identified by comparing the similarity of the mass spectra and retention time with the standard solutions and with the literature. The following standard compounds were evaluated: gallic acid, caffeic acid, ferulic acid, rosmarinic acid, chlorogenic acid, apigenin, rutin and quercetin.

### DPPH radical scavenging assay

The DPPH scavenging activity of the leaf and bark extracts was evaluated by bleaching of the purple methanol solution of free radical DPPH according to Scherer and Godoy (2009). Antioxidant activity was expressed as IC_50_ value (μg/mL) and by the antioxidant activity index (AAI). The assays were carried out in triplicate and antioxidant activity was compared with the commonly used chlorogenic acid and tocopherol (vitamin E).

### Ferric reducing antioxidant power assay (FRAP)

Antioxidant capability of *S. australis* extract was estimated as described by Pulido et al. (2000) with modifications. FRAP reagent (270 μL), freshly prepared, was mixed with 30 μL of test sample or ethanol (for the reagent blank). The test samples and reagent blank were incubated at room temperature for 10 min. The FRAP reagent contained 2.5 mL of 2,4,6-tripyridyl-2-triazine (TPTZ) solution in 40 mM HCl plus 2.5 mL of 20 mM FeCl_3_·6H_2_O and 25 mL of 0.3 M acetate buffer (pH 3.6). At the end of incubation, the absorbance was measured at 595 nm using a microplate reader (Molecular Devises, Spectra Max 190, Sunnyvale, CA). Ethanolic solutions of known Fe II concentration were used for the preparation of the calibration curve. The FRAP value was expressed as mmol Fe (II) equivalent/mg extract. The experiments were carried out at least in triplicate.

### Nitric oxide radical scavenging assay

Nitric oxide radical (NO^−^) generated from sodium nitroprusside (SNP) was measured using the Griess reaction. Briefly, the reaction mixture containing sodium nitroprusside (10 mM) in phosphate buffered saline (pH 7.3) with or without the plant extract at different concentrations (62.5–1000.0 μg/mL) was incubated at room temperature for 30 min in a 96 wells plate. Next, 150 μL of incubated solution was mixed with 150 μL of Griess reagent (1% sulfanilamide in 5% H_3_PO_4_ and 0.1% *N*-(1-naphthyl) ethylenediamine in distilled water in equal volumes) and the absorbance of chromophore formed during the diazotization of nitrite ions with sulfanilamide and subsequent coupling with naphthylethylene-diaminedihydrochloride was measured at 540 nm in an ELISA plate reader (Molecular Devices Spectra MAX 190 Orleans Drive Sunnyvale, CA). Gallic acid was used as positive control. The quantification of nitrite was calculated by regression analysis from a standard curve of sodium nitrite and the percentage of the NO inhibition was calculated by using the nitrite level of SNP-induced group as control.

### Measurement of cell viability

Cell viability studies were performed using the MTT assay. Macrophages RAW 264.7, 3T3 fibroblasts and Hepa 1c1c7 cells were plated at a density of 7 × 10^5^ cells/mL in 96-well flat-bottomed tissue culture plates. After overnight incubation, the cells were incubated for additional 24 h in the presence or absence of increasing concentrations (10.0–500.0 μg/mL) of the ethanol leaf and barks extracts. Camptothecin was used as the positive control. After incubation, 100 μL of 3-(4,5-dimethylthiazol-2-yl)-2,5-diphenyl tetrazolium bromide (MTT) (1 mg/mL) was added per well, and the plate was incubated for 2 h. The formazan crystals formed by cellular mitochondrial dehydrogenases were then dissolved with dimethyl sulfoxide (DMSO) cells. The optical density of purple formazan, proportional to the number of viable cells, was measured at 595 nm using a microplate spectrophotometer (SpectraMax 190; Molecular Devices, Sunnyvale, CA). Experiments were carried out at least in triplicate.

### Nitric oxide analysis in the supernatant of macrophage cell culture

RAW 264.7 cells were plated at 5.0 × 10^5^ cells/mL in 24-well tissue culture plates and incubated with or without ethanol leaf and bark extracts of *S. australis* (2.0–50.0 μg/mL) for 2 h. Next, cells were stimulated with LPS (1 μg/mL)/IFN-γ (10 ng/mL) and incubated for additional 24 h. Nitrite accumulation in the culture medium as an indicator of NO production was measured using the Griess reagent (1% sulfanilamide in 5% H_3_PO_4_ and 0.1% *N*-(1-naphthyl)ethylenediamine in distilled water) (Green et al. 1982). The culture supernatant (100 mL) was mixed with 100 mL of Griess reagent and incubated for 10 min. The absorbance at 540 nm was measured in an ELISA plate reader (SpectraMax 190; Molecular Devices, Sunnyvale, CA) and the inhibitory rates were calculated by using a standard calibration curve prepared with sodium nitrite, by comparing with the LPS/IFN-γ stimulated control group.

#### Measurement of TNF-α

Macrophage RAW 264.7 cells were exposed to LPS (1 μg/mL)/IFN-γ (10 ng/mL) with or without ethanol leaf and bark extracts of *S. australis* (10.0–100.0 μg/mL) for 24 h. Next, 100 μL of the culture supernatant was used to determine the level of TNF-α by EIA assay kit technique using specific antibodies and cytokine standards according to the manufacturer’s instructions (eBioscience, San Diego, CA).

### Nuclear factor-κB luciferase assay

Human embryonic kidney cells 293 (Panomic, Fremont, CA) were used for monitoring changes occurring along the NF-κB pathway (Kondratyuk et al. 2012). Stable constructed cells were seeded into 96-well plates at 20 × 10^3^ cells/well. Cells were maintained in Dulbecco’s modified Eagle’s medium (DMEM) (Invitrogen Co.; Carlsbad, CA), supplemented with 10% fetal bovine serum (FBS), 100 units/mL penicillin, 100 μg/mL streptomycin and 2 mM l-glutamine. After 48 h of incubation, the medium was replaced and the cells were treated with various concentrations of test compounds. TNF-α (Human Recombinant, *E. coli*, Calbiochem, Gibbstown, NJ) was used as an activator at a concentration of 2 ng/mL (0.14 nM). The plate was incubated for 6 h. Spent medium was discarded and the cells were washed once with PBS. Cells were lysed using 50 μL (for 96-well plate) of Reporter Lysis Buffer from Promega, by incubating for 5 min on a shaker, and stored at −80 °C. The luciferase assay was performed using the Luc assay system from Promega (Madison, WI). The gene product, luciferase enzyme, reacts with luciferase substrate, emitting light which was detected using a luminometer (Multi-Mode Microplate Reader, Filter Max F5, Molecular Devices, Sunnyvale, CA). Data for NF-κB constructs were expressed as IC_50_ values (i.e. the concentration required to inhibit TNF-activated NF-κB activity by 50%). As a positive control, two known NF-κB inhibitors were used: TPCK, IC_50_ 3.8 μM and BAY-11, IC_50_ 2.0 μM.

### Antibacterial activity

The antibacterial properties of the extracts were evaluated using the standard NCCL method broth dilution method (NCCL, 2008), in a 96-well microtiter plate. The minimum inhibitory concentrations (MICs) were determined against the Gram-positive bacteria *Staphylococcus aureus* (ATCC 25923) and *Streptococcus agalactiae* (ATCC 12386), and the Gram-negative bacteria *Escherichia coli* (ATCC 8739), *Salmonella typhimurium* (ATCC 14028) and *Klebsiella pneumoniae* (CCCD K003), and yeast represented by *Candida albicans* (ATCC 10231). The final concentration of cells was adjusted in a turbidimeter (930 NTU – Nephelometric Turbidity Units) 0.5 on the McFarland scale, in the order of 10^6^ CFU/ml. 100 μL of culture medium (Mueller–Hinton broth 2.1%), sample or antibiotic, and the inoculum were added to each well. The final tested concentrations of the extracts were 1000.0, 500.0, 250.0, 125.0 and 62.5 μg/mL. After the addition of inoculum, the plates were incubated for 24 h. Later on, 100 μL of triphenyl tetrazolium chloride (TTC) (0.5% aqueous solution) was added. After 4 h incubation, the MIC was determined as the lowest concentration capable of inhibiting visible growth of cells, checked by TTC. In all plates, positive and negative controls (six wells of each) were included. The experiments were carried out at least in triplicate.

### Statistical analysis

Statistical analysis was performed using the software GraphPad Prism 5 (San Diego, CA). The results are expressed as the mean ± standard error of mean (SEM) or standard deviation (SD) of triplicate experiments. Statistically significant values were compared using a using one- or two-way analysis of variance (ANOVA), with the Tukey test for *post hoc* comparisons. Values of *p* < 0.05 was considered significant.

## Results

Determination of total phenolic (TPC) and tannin content in the leaf and bark extracts of *S. australis* revealed that the medicinal plant is rich in phenolic compounds (Table 1). TPC were expressed as milligram equivalents of pyrogallol per gram of crude extract. Higher amounts of TPC was detected in the leaves compared to the bark (395.24 ± 3.97 and 381.35 ± 1.98 mg/g of pyrogallol, respectively), although the difference was not significant (*p* > 0.05). Tannin content in the extracts ranged from 47.62 ± 1.75 to 77.38 ± 1.98 mg/g of dry extract in the leaf and bark extracts, respectively, with significant differences (*p* < 0.05) (Table 1).

**Table 1.** Quantification of total phenolics and tannins content present in the ethanol leaf and bark extracts of *S. australis*.

Plant material	Total phenolic*(mg/g)	Tannins*(mg/g)
Ethanol leaf extract	395.24 ± 3.97^a^	77.38 ± 1.98^a^
Ethanol bark extract	381.35 ± 1.98^a^	47.62 ± 1.75^b^

Different letters in the same column correspond to significant differences (*p* < 0.05). Tests was performed in triplicate and expressed as mean ± standard error.

* Results expressed in mg of pyrogallol equivalents per gram of crude extract.

**Figure 1.**
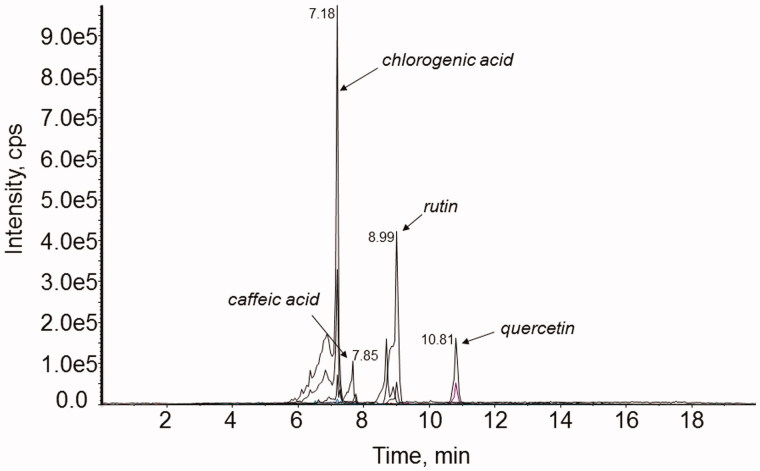
Representative chromatogram of the ethanol leaf extract of *S. australis* (under conditions described in LC-ESI-MS/MS analyses).

The major constituents in the ethanol leaf extract were identified using LC-MS/MS analyses. Figure 1 shows a representative chromatogram of the ethanol leaf extract of *S. australis*, exhibiting the presence of phenolic compounds caffeic acid and chlorogenic acid and the flavonoids rutin and quercetin as major compounds.

Natural bioactive compounds such as phenols are described as important secondary metabolites with intrinsic properties that affect oxidative stability and present many biological properties, including antioxidant activity (Singh et al. 2012). DPPH scavenging activity was expressed as IC_50_ in μg/mL and by Antioxidant Activity Index (AAI) proposed by Scherer and Godoy (2009), which classifies the antioxidants as weak when AAI <0.5, moderate when AAI is between 0.05 and 1.0, strong when AAI is between 1.0 and 2.0, and very strong when AAI >2.0. The ethanol leaf and bark extract exhibited moderate activity, with AAI of 0.8 ± 0.03 and 0.5 ± 0.03, respectively. Tocopherol presents very strong activity (AAI of 3.2 ± 1.10) (Table 2). The ferric reducing antioxidant power (FRAP) is a simple and rapid method, and is based on the reduction of the Fe^3+ ^complex of tripyridyltriazine (Fe(TPTZ)^3+^) to the intensely blue-coloured Fe^2+ ^complex (Fe(TPTZ)^2+^) by antioxidants (Russo et al. 2015). The ethanol leaf and bark extracts showed a weak ferric reducing antioxidant power, with an IC_50_ of 312.6 ± 2.61 μg/mL and 568.3 ± 4.72 μg/mL, respectively. Quercetin (used as standard) exhibited an IC_50_ of 15.7 ± 0.32 μg/mL (Table 2). Sodium nitroprusside spontaneously generates NO as free radical in aqueous solution at physiological pH (7.2) which interacts with oxygen to produce nitrite ions that can be estimated by the Griess reaction. The scavenging effects of ethanol leaf and bark extracts of *S. australis* on NO production was estimated using sodium nitroprusside, which spontaneously generates NO as free radical in aqueous solution at physiological pH (Boora et al. 2014). The results expressed as IC_50_ are shown in Table 2. The ethanol leaf extract (IC_50_ of 285.0 ± 9.61 μg/mL) exhibited interesting NO scavenging activity, comparable with the triphenolic compound gallic acid used as reference (IC_50_ value of 223.2 mg/mL ±4.36 μg/mL). The bark extract exhibited only week scavenging activity, with an IC_50_ value of 972.6 ± 12.15 μg/mL.

**Table 2.** *In vitro* antioxidant activity of ethanol leaf and bark extract of *S. australis* determined by DPPH radical scavenging activity, ferric reducing antioxidant power (FRAP) and nitric oxide radical scavenging assay.

	DPPH scavenging activity		FRAP	NO radical Scavenging activity
Sample	IC_50_ (μg/mL)	AAI	IC_50_ (μg/mL)	IC_50_ (μg/mL)
ELE	43.5 ± 1.55^a^	0.8 ± 0.03^a^	312.6 ± 2.61^a^	285.0 ± 9.61^a^
EBE	66.2 ± 0.78^a^	0.5 ± 0.03^a^	568.3 ± 4.72^b^	972.6 ± 12.15^b^
Tocopherol	11.8 ± 6.30^b^	3.2 ± 1.10^b^	–	–
Quercetin	–	–	15.7 ± 0.32^c^	–
Gallic acid	–	–	–	223.2 ± 4.36^a^

ELE: ethanol leaf extract; EBE: ethanol bark extract. Different letters in the same column correspond to significant differences (*p* < 0.05). Tests (*n* = 3) were performed in triplicate and expressed as mean ± standard error.

The cytotoxic activities of extracts was tested *in vitro* against one normal cell line (mouse fibroblasts) and two cancer cell lines: the human ovarian carcinoma cell line (OVCAR-3) and murine (Hepa 1c1c7) hepatoma cells. No cytotoxic effects were observed for the leaf and bark extracts at concentrations ranging from 1.0 to 100.0 μg/mL (data not shown) in all tested cell lines.

The antimicrobial activity of *S. australis* leaf and bark ethanol extract was tested against five different bacterial strains and the fungus *C. albicans*, and indicated variable activities against all the organisms tested as shown in Table 3. The minimum inhibitory concentration (MIC) evidenced that the ethanol leaf and bark extracts of *S. australis* exhibited promising antibacterial activity against *Salmonella* and *Klebsiella* with an MIC value of 250 μg/mL. The ethanol leaf extract also shows weak activity against the Gram positive bacteria *S. aureus* (MIC 1000 μg/mL) and the fungus *C. albicans* (MIC 500 μg/mL).

**Table 3.** Antimicrobial activity of the ethanol leaf and bark extracts of *S. australis* expressed as Minimum Inhibitory Concentration (MIC) in μg/mL.

	MIC (μg/mL)			
	Ethanol leaf extract	Ethanol bark extract	Penicillin	Norfloxacin
*S. aureus*	1000	>1000	–	500
*S. agalactiae*	>1000	>1000	100	–
*C. albicans*	500	1000	–	30
*E. coli*	>1000	>1000	100	250
*S. typhimurium*	250	250	100	–
*K. pneumoniae*	250	250	100	–

**Figure 2.**
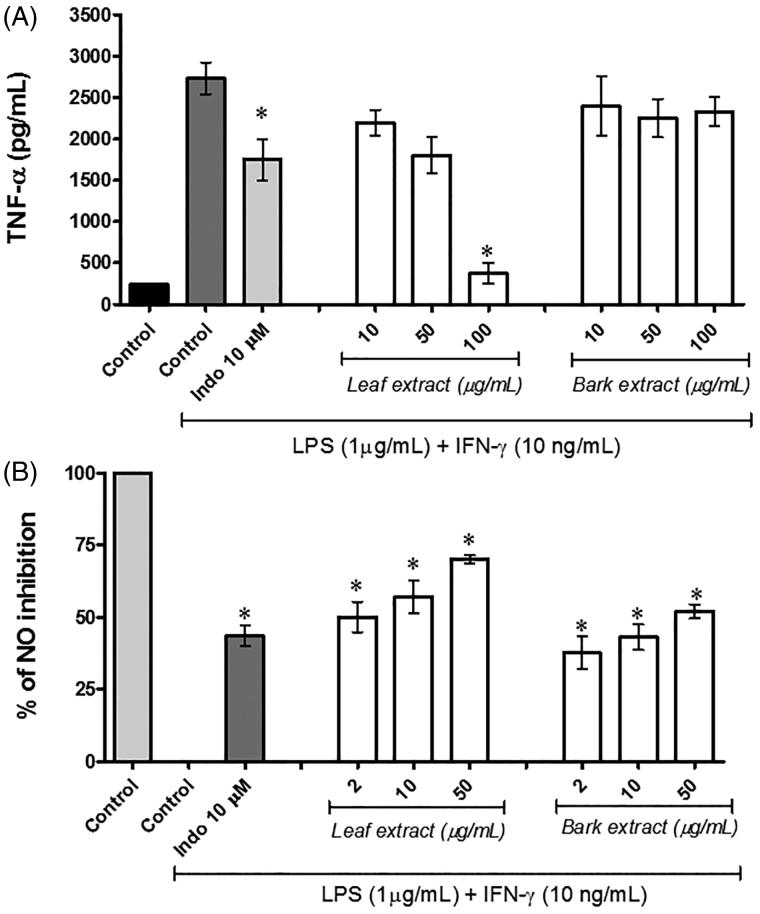
*S. australis* reduce nitric oxide (NO) and TNF-α concentration *in vitro*. RAW 264.7 macrophage were exposed with or without ethanol leaf and barks extracts of *S. australis* for 2 h and then stimulated with LPS/IFN-γ. (A) TNF-α and (B) NO production was measured 24 h later using ELISA Kit and the Griess reagent, respectively, as described in the “Materials and methods” section. Results are expressed as mean ± SD of three independent experiments. *Significant at *p* < 0.05 compared to control LPS/IFN-γ-induced cells.

Concerning the cytokines production, the effects of ethanol leaf and bark extracts of *S. australis* on LPS/IFN-γ induced inflammation in RAW 264.7 macrophage were evaluated by measuring the production of NO and pro-inflammatory cytokines. As observed in Figure 2, stimulation with LPS/IFN-γ for 24 h significantly induced the release of NO and TNF-α indicating that an inflammatory response was induced in the RAW 264.7 cells. As shown in Figure 2(A), when RAW 264.7 cells were treated with the ethanol leaf extract of *S. australis* at 100 μg/mL, a significant inhibition of TNF-α production was observed (*p* < 0.05). Ethanol bark extract did not reduce TNF-α production in RAW 264.7 cells. As shown in Figure 2(B), both extracts caused a significant decreased in NO release compared to the LPS/IFN-γ control group (*p* < 0.05). The leaf extract exhibited higher inhibition effect in all tested concentrations, reaching 70% at a concentration of 50.0 μg/mL.

The inhibitory effects of the ethanol leaf extract of *S. australis* on TNF-α and NO production prompted us to further investigate its ability to influence the nuclear factor (NF)-κB signalling pathway. The suppression of NF-κB activation by the ethanol leaf extract of *S. australis* was 12.8 ± 2.6% and 20.4 ± 3.5% at concentrations of 50 and 100 μg/mL, respectively.

## Discussion

Different plant extracts and single compounds isolated from the *Sambucus* genus were reported to have relevant pharmacological activities, such as anti-inflammatory (Schwaiger et al. 2011), antibacterial (Salehzadeh et al. 2014) and wound healing activity (Süntar et al. 2010). However, despite being widely used in folk medicine, the biological activities of the species *S. australis* has not been investigated (Lorenzi & Matos 1985; Brandão et al. 2006; Nunes et al. 2007). In this study, for the first time, the authors show that the ethanol leaf extract of *S. australis* was able to exert anti-inflammatory effects in LPS/IFN-γ activated RAW 264.7 macrophage which may be related, at least in part, to the inhibitory activity of the pro-inflammatory mediators, NO and TNF production and suppression of NF-κB activation.

Inflammation is one of the most important biological protective responses to tissue injury or microbial invasion, and is capable of causing cell injury, under which pro-inflammatory mediators are released (Sacca et al. 1997; Serhan et al. 2007). Inflammatory factors are considered fundamental elements in the chronic inflammation associated with many diseases, such as atherosclerosis, obesity, diabetes, neurodegenerative diseases and cancer. Steroidal and non-steroidal anti-inflammatory drugs are currently used to treat acute inflammation. However, these drugs are not entirely successful in curing chronic inflammatory disorders, and often have side effects (Lichtenstein et al. 1995; Mckellar et al. 2007). Therefore, the identification of new, safer and more effective anti-inflammatory compounds is necessary (Cragg & Newman 2013; Russo et al. 2015). Currently, great attention has been devoted to the use of natural compounds, especially phenolic compounds, which are well-known phytochemicals found in all plants and have notable pharmacological properties, including antioxidant activity (Singh et al. 2012; Barbaro et al. 2014; Kim et al. 2014). In the present study, especially, leaf extract of *S. australis* exhibited an expressive amount of phenolic compounds, with caffeic acid and chlorogenic acid being the major compounds, and a moderate concentration of total tannins. The antioxidant activity estimated using different *in vitro* antioxidant tests exhibited by the leaf extract could be associated with the significant phenolic content, suggesting a correlation between these constituents and antioxidant activity. These results are in agreement with the current literature, which emphasizes the relationship between antioxidant activity and the presence of phenolic compounds, showing that they are responsible for quenching different free radicals (Al-Zoreky 2009; Hossain et al. 2011; Saeed et al. 2012; Xu et al. 2015).

Nitric oxide (NO) is a free radical with important complex regulatory activity on the functions, growth and death of many cell types involved in immune and inflammatory responses (Sharma et al. 2016). NO is produced through the action of iNOS, and is present at low levels under normal physiological conditions; however, it is rapidly induced by pro-inflammatory and mitogenic stimuli, including LPS (Coleman 2001). Many studies have demonstrated that excessive NO production plays a critical role in the pathogenesis of inflammation, and can lead to tissue damage by reacting with reactive oxygen species (Martinon 2010; Harijith et al. 2014). On the other hand, inhibitors of NO induction have been reported to exert anti-inflammatory effects by preventing iNOS expression (Achoui et al. 2010, Pinho et al. 2011; Rebelo et al. 2014). In the present study, the obtained *in vitro* results showed that *S. australis* significantly reduced NO production in LPS/IFN-γ stimulated RAW 264.7 macrophages, and possesses good NO radical scavenging activity leading to the reduction of the nitrite concentration generated from sodium nitroprusside. This suggests that inhibition of NO contributes to the anti-inflammatory activity of *S. australis*, without affecting the viability of these cells.

Inflammatory disorders are also characterized, among other events, by the production and release of pro-inflammatory cytokines such as tumour necrosis factor-α (Carballo et al. 1998). TNF-α is considered a critical cytokine in the inflammatory cytokine network, and is important for promoting the expression of iNOS and production of other cytokines. Therefore, suppressing the overproduction and activity of pro-inflammatory cytokines is necessary to reduce inflammation and its symptoms, and this method has proved to be successful in the treatment of certain inflammatory diseases (Fullerton & Gilroy 2016; Lai & Dong 2016). In these study, it was demonstrated that LPS/IFN-γ-induced production of TNF-α was significant affected by the ethanol leaf extract of *S. australis*, suggesting the potential anti-inflammatory activity of the extract. Corroborating with our results, the current literature has described the potential anti-inflammatory activity of *Sambucus* genus by inhibiting macrophage release of pro-inflammatory cytokines including TNF-α (Harokopakis et al. 2006; Schwaiger et al. 2011).

Of the several transcriptional factors activated by inﬂammatory responses, NF-κB is known to induce the transcription of pro-inﬂammatory mediators, such as inducible NO synthase (iNOS), cyclooxygenase (COX)-2, TNF-α, IL-1, and IL-6 (Surh et al. 2001). Thus, we considered that the NF-κB signalling pathway might be involved in the ethanol leaf extract mediated down-regulations of NO and TNF-α. Therefore, we investigated the effects of ethanol leaf extract of *S. australis* on the NF-κB signalling pathway and demonstrated that the molecular mechanism by which the ethanol leaf extract of *S. australis* inhibits the expression of these inﬂammatory mediators appeared to be only partially involved the inhibition of NF-κB activation. The search for new chemotherapeutic alternatives from traditional medicine led to great success in eliminating infections caused by drug-resistant microbes, and reducing harm caused by antibiotics (Sharma et al. 2016). MIC values obtained from leaf and bark extracts exhibited prominent antibacterial activity against Gram negative bacteria, especially *Salmonella typhimurium* and *Klebsiella pneumoniae*. According to Alberto et al. (2001), phenolic compounds may affect the growth and metabolism of bacteria. They may have distinct effects by activating or inhibiting microbial growth according to their constitution and concentration. Thus, the antibacterial activity exhibited by *S. australis* extracts could be associated to the significant phenolic content present in the leaves and bark. Corroborating with our results, previous studies have found that *S. ebulus* extracts were active against *Staphylococcus aureus* and *Pseudomonas aeruginosa* (Ghesmati 2008); *S. nigra* flower exhibited strong antimicrobial effects on various nosocomial pathogens, notably methicillin-resistant *S. aureus* (Hearst et al. 2010).

## Conclusions

The present investigation suggests that *S. australis* extracts have the ability to inhibit the production of pro-inflammatory mediators such as NO and TNF-α in LPS/IFN-γ stimulated macrophages, an action that is only partially dependent on NF-κB signalling pathway; they possess free radical scavenging activity, and provide protection against microbial infections. These biological properties may be attributed to the potential of different constituents, especially polyphenols. In conclusion, these preliminary *in vitro* results are encouraging for further biological and phytochemical studies aimed at isolating and identifying the active principles, which could provide scientific evidence for the popular use of *S. australis* and contribute to the development of new therapeutic strategies against inflammatory disorders.
